# Bevacizumab beyond Progression for Newly Diagnosed Glioblastoma (BIOMARK): Phase II Safety, Efficacy and Biomarker Study

**DOI:** 10.3390/cancers14225522

**Published:** 2022-11-10

**Authors:** Motoo Nagane, Koichi Ichimura, Ritsuko Onuki, Daichi Narushima, Mai Honda-Kitahara, Kaishi Satomi, Arata Tomiyama, Yasuhito Arai, Tatsuhiro Shibata, Yoshitaka Narita, Takeo Uzuka, Hideo Nakamura, Mitsutoshi Nakada, Yoshiki Arakawa, Takanori Ohnishi, Akitake Mukasa, Shota Tanaka, Toshihiko Wakabayashi, Tomokazu Aoki, Shigeki Aoki, Soichiro Shibui, Masao Matsutani, Keisuke Ishizawa, Hideaki Yokoo, Hiroyoshi Suzuki, Satoshi Morita, Mamoru Kato, Ryo Nishikawa

**Affiliations:** 1 Department of Neurosurgery, Kyorin University Faculty of Medicine, Tokyo 181-8611, Japan; 2 Department of Brain Disease Translational Research, Juntendo University Graduate School of Medicine, Tokyo 113-8421, Japan; 3 Division of Bioinformatics, National Cancer Center Research Institute, Tokyo 104-0045, Japan; 4 Division of Brain Tumor Translational Research, National Cancer Center Research Institute, Tokyo 104-0045, Japan; 5 Department of Diagnostic Pathology, National Cancer Center Hospital, Tokyo 104-0045, Japan; 6 Department of Brain Disease Translational Research, Juntendo University Faculty of Medicine, Tokyo 113-8421, Japan; 7 Division of Cancer Genomics, National Cancer Center Research Institute, Tokyo 104-0045, Japan; 8 Department of Neurosurgery and Neuro-Oncology, National Cancer Center Hospital, Tokyo 104-0045, Japan; 9 Department of Neurosurgery, Dokkyo Medical University, Tochigi 321-0293, Japan; 10 Department of Neurosurgery, Faculty of Life Sciences, Kumamoto University, Kumamoto 860-8555, Japan; 11 Department of Neurosurgery, Graduate School of Medical Sciences, Kanazawa University, Kanazawa 920-1192, Japan; 12 Department of Neurosurgery, Graduate School of Medicine, Kyoto University, Kyoto 606-8501, Japan; 13 Department of Neurosurgery, Graduate School of Medicine, Ehime University, Ehime 790-0052, Japan; 14 Department of Neurosurgery, Graduate School of Medicine, The University of Tokyo, Tokyo 113-8654, Japan; 15 Department of Neurosurgery, Graduate School of Medicine, Nagoya University, Aichi 464-8601, Japan; 16 Department of Neurosurgery, Kyoto Medical Center, Kyoto 612-8555, Japan; 17 Department of Radiology, Graduate School of Medicine, Juntendo University, Tokyo 113-8421, Japan; 18 Department of Neurosurgery, Teikyo University Hospital, Kawasaki 213-8507, Japan; 19 Department of Neurosurgery, Kurosawa Hospital, Gunma 370-1203, Japan; 20 Department of Pathology, Saitama Medical University, Saitama 350-0495, Japan; 21 Department of Human Pathology, Graduate School of Medicine, Gunma University, Gunma 371-8511, Japan; 22 Department of Pathology and Laboratory Medicine, National Hospital Organization Sendai Medical Center, Miyagi 983-8520, Japan; 23 Department of Biomedical Statistics and Bioinformatics, Graduate School of Medicine, Kyoto University, Kyoto 606-8501, Japan; 24 Department of Neuro-Oncology/Neurosurgery, Saitama Medical University International Medical Center, Saitama 350-1298, Japan

**Keywords:** bevacizumab, glioblastoma, temozolomide, progression, biomarker

## Abstract

**Simple Summary:**

This was a multicenter, single-arm, phase II study comprising two protocol treatments. Patients were enrolled after craniotomy or biopsy and initiated the concurrent phase; oral daily temozolomide concomitant with radiation therapy during the first 6 weeks of treatment. Bevacizumab was intravenously administered every other week. The protocol-defined secondary therapy (i.e., BBP regimen) was given as bevacizumab monotherapy or in combination with other chemotherapeutic agents upon first progression or recurrence until further progression or unacceptable toxicity developed. The primary endpoint, the 2-year survival rate of the BBP group, was 27.0% and was unmet. Expression profiling using RNA sequencing identified that Cluster 2, enriched with the genes involved in macrophage or microglia activation, was associated with longer OS and PFS independent of the *MGMT* methylation status.

**Abstract:**

We evaluated the efficacy and safety of bevacizumab beyond progression (BBP) in Japanese patients with newly diagnosed glioblastoma and explored predictors of response to bevacizumab. This phase II study evaluated a protocol-defined primary therapy by radiotherapy with concurrent and adjuvant temozolomide plus bevacizumab, followed by bevacizumab monotherapy, and secondary therapy (BBP: bevacizumab upon progression). Ninety patients received the protocol-defined primary therapy (BBP group, *n* = 25). Median overall survival (mOS) and median progression-free survival (mPFS) were 25.0 and 14.9 months, respectively. In the BBP group, in which *O^6^*-methylguanine-DNA methyltransferase (*MGMT*)-unmethylated tumors predominated, mOS and mPFS were 5.8 and 1.9 months from BBP initiation and 16.8 and 11.4 months from the initial diagnosis, respectively. The primary endpoint, the 2-year survival rate of the BBP group, was 27.0% and was unmet. No unexpected adverse events occurred. Expression profiling using RNA sequencing identified that Cluster 2, which was enriched with the genes involved in macrophage or microglia activation, was associated with longer OS and PFS independent of the *MGMT* methylation status. Cluster 2 was identified as a significantly favorable independent predictor for PFS, along with younger age and methylated *MGMT*. The novel expression classifier may predict the prognosis of glioblastoma patients treated with bevacizumab.

## 1. Introduction

Glioblastoma (GBM), the most common primary brain tumor among adults, is an aggressive glioma with a poor prognosis [1] and recurrence in most patients [2]. Although the current standard of care for GBM involves surgical resection followed by radiotherapy with concurrent and adjuvant temozolomide (Stupp regimen) [3], the median progression-free survival (mPFS) is only 6.9 months, and median overall survival (mOS), 14.6 months [3]. *O6*-methylguanine-DNA methyltransferase (*MGMT*) promoter methylation is a strong prognosis factor and temozolomide response predictor for GBM [4,5,6].

Bevacizumab is a humanized monoclonal antibody against vascular endothelial growth factor A (VEGFA). VEGFA (known as VEGF) is the major angiogenic factor for tumor angiogenesis [7]. Therefore, an anti-VEGF antibody is expected to benefit patients with highly angiogenetic tumors, such as GBM [8,9,10]. Although several bevacizumab studies have been conducted in patients with newly diagnosed and recurrent GBM [11,12,13,14], evidence supporting an effective bevacizumab GBM regimen has been insufficient. Prolonged PFS without prolonged OS has been reported in patients with both newly diagnosed and recurrent GBM [11,12,15,16].

A new regimen, bevacizumab beyond progression (BBP), comprises the extended use of bevacizumab, added to second-line chemotherapy upon progression in unresectable, advanced, recurrent cancer, leading to OS prolongation in colorectal and breast cancers [17,18]. In highly angiogenic GBM, tumor cells may continuously produce VEGF even at recurrence and promote further angiogenesis. Bevacizumab discontinuation at disease progression may therefore result in acute tumor progression [19], which is considered to be one of the reasons for the extremely poor prognosis of patients, given that no effective second-line standard therapies have been developed yet. A retrospective pooled analysis of phase II studies suggested that PFS and OS were significantly improved in the BBP group compared with the non-BBP group [20]. However, two prospective phase II studies of BBP (TAMIGA and CABARET Part 2) did not show clear survival improvements [21,22].

Two previous phase III studies reported clinically inconsistent results [11,12]. This suggests there may be a subgroup in the GBM population in which bevacizumab could be more effective than others [11,21,22]. However, biomarkers to predict bevacizumab efficacy have not been thoroughly investigated. A retrospective analysis of the AVAglio study showed OS benefits for patients with isocitrate dehydrogenase 1 (*IDH1*) WT proneural GBM when bevacizumab was combined with the standard regimen [23]. This observation suggested that there may be a subset of patients with GBM who could benefit from continuous administration of bevacizumab beyond progression. 

This study (BIOMARK) evaluated the efficacy and safety of BBP in patients with newly diagnosed GBM after surgery. We conducted a thorough genomic analysis to investigate potential biomarkers to identify the subpopulations that may benefit most from BBP or bevacizumab first-line treatment.

## 2. Materials and Methods

### 2.1. Study Design

This was a multicenter, single-arm, phase II study comprising two protocol treatments: protocol-defined primary therapy (comprising concurrent, maintenance and monotherapy phases) and protocol-defined secondary therapy. Patients were enrolled within 7 to 21 days after craniotomy or biopsy and initiated the concurrent phase within the next 3 weeks; oral daily temozolomide (75 mg/m^2^ per day) concomitant with radiation therapy (60 Gy: 2-Gy fractions 5 days/week) during the first 6 weeks of treatment. Bevacizumab was intravenously administered on Day 1 of Weeks 4, 6, and 8 at 10 mg/kg per dose (Figure S1). 

In the maintenance phase, combination therapy with oral temozolomide (150 to 200 mg/m^2^ per day on Days 1–5 every 4 weeks) and intravenous bevacizumab (10 mg/kg, on Days 1 and 15 of each cycle) was provided for up to twelve 4-week cycles (48 weeks), unless exacerbation or recurrence was observed. If temozolomide was discontinued during the concurrent or maintenance phase, the monotherapy phase was started from discontinuation. In the monotherapy phase, intravenous bevacizumab was administered at 10 mg/kg per dose in 2-week cycles or 15 mg/kg per dose in 3-week cycles until progression or recurrence was observed.

The protocol-defined secondary therapy (i.e., BBP regimen) was given as bevacizumab monotherapy or in combination with other chemotherapeutic agents upon first progression or recurrence. Bevacizumab was administered in 2-week or 3-week cycles until further progression or unacceptable toxicity developed, using the same bevacizumab dose as in the monotherapy phase.

### 2.2. Patients

Patients were eligible if they were aged 20–75 years; had newly diagnosed, histologically confirmed supratentorial GBM, Grade IV, by World Health Organization Classification of Tumours of the Central Nervous System, revised 4th Ed (the diagnosis was based on the WHO Classification at the time when the study was designed, see Results), without dissemination or gliomatosis cerebri; had an available surgical specimen (including fresh frozen specimen); had a Karnofsky Performance Status (KPS) ≥ 60; had adequate hematologic, hepatic, and renal function after surgery, and could provide informed consent. Patients with MRI-confirmed new bleeding after cranial surgery; history of chemotherapy, radiotherapy, or immunotherapy (including vaccines); or uncontrolled hypertension, history of stroke or unstable angina, myocardial infarction, intracranial abscess within 6 months before randomization, or a serious nonhealing wound were excluded.

### 2.3. Study Endpoint

The primary outcome (BBP efficacy) was the 2-year survival rate in patients who received at least one protocol-defined secondary therapy (defined as the BBP group). This endpoint was adopted from the 2-year survival rate in AVAglio, which was approximately 30% in both arms, providing an adequate margin for evaluation of the add-on effect of BBP. Secondary outcomes included the 2-year survival rate and OS among patients who received at least one protocol-defined therapy (full analysis set [FAS]), PFS, objective response rate (FAS), and safety. Quality of life and neurocognitive functions were also evaluated. Adverse event (AE) data were collected and reported using Common Terminology Criteria for Adverse Events version 4.0. Patients who did not have a confirmed cytological or histopathological GBM by central pathological review and had any efficacy data after starting protocol-defined therapies were excluded from the evaluations.

### 2.4. Biomarker Analysis

#### 2.4.1. Biomarker Analysis Cohort

The entire FAS cohort except for one case for which tumor tissue was unavailable (89 cases) and 19 cases from the placebo-controlled group of the AVAglio study were subjected to the biomarker analysis. Fresh frozen surgical tumor specimens were available for all biomarker analysis cohort patients. Mutation analysis for *IDH1*/*IDH2* and the *TERT* promoter by pyrosequencing, targeted sequencing by Ion Proton, *MGMT* methylation analysis by pyrosequencing, and the genome-wide DNA methylation analysis by the EPIC array were performed in all cases. RNA sequencing and the NanoString analysis were performed in the cases where the quality of RNA was sufficient for each analysis (Table S1). Six patients had *IDH1* R132H mutation (three patients in the FAS and three in AVAglio). These cases were excluded from further biomarker analysis.

#### 2.4.2. Histopathological Review and Tumor Cell Content Estimation

The histopathological diagnoses of all patients were reviewed according to the revised 4th edition of the WHO Classification of Tumours of the Central Nervous System by consensus of three board-certified pathologists (KI, HY, HS) [24]. For tumor cell content estimation, a portion of the fresh frozen tumor specimen subjected to the biomarker analysis was formalin-fixed and paraffin-embedded. The entire area of hematoxylin–eosin stained slides was visually inspected by a single board-certified pathologist (KS), and the percentage of tumor cell contents and necrotic fractions were estimated in each case by microscope.

#### 2.4.3. DNA/RNA Extraction

DNA was extracted from the frozen tumor tissues using a DNeasy Blood & Tissue Kit (Qiagen, Tokyo, Japan). Total RNA was extracted from the frozen tumor tissues using an miRNeasy Micro Kit (Qiagen, Tokyo, Japan). 

#### 2.4.4. IDH1/2 Mutation/TERT Promoter Mutation/MGMT Promoter Methylation Analysis

The presence of the hotspot mutations in *IDH1*, *IDH2,* and the *TERT* promoter was assessed by pyrosequencing for all cases enrolled in the study as previously described [25]. The methylation status of the *MGMT* promoter was analyzed by pyrosequencing after bisulfite modification of genomic DNA extracted from tumor specimens as described [25]. Based on an outcome-based study to determine an optimal cutoff to judge *MGMT* promoter methylation in a series of 276 newly diagnosed GBMs, we used a cutoff of ≥ 16% for *MGMT* methylation. The details of this study will be described elsewhere (Ichimura, manuscript in preparation).

#### 2.4.5. Targeted Sequence by Ion Proton

Target sequencing for all coding exons of 93 genes known to be frequently mutated in brain tumors was performed using an Ion Proton Sequencer and the Ion Chef System (Thermo Fisher Scientific, Tokyo, Japan) according to the manufacturer’s instruction as previously described [26]. Reads were mapped onto the hg19 human reference genome sequence, and variant call was performed using Ion Reporter software (Thermo Fisher Scientific, https://ionreporter.thermofisher.com/ir/ (accessed on 10 April 2019)). UCSC Common SNPs were excluded.

#### 2.4.6. RNA Sequencing

RNA sequencing was essentially performed as described previously [27]. Briefly, total RNA was quantified using Qubit RNA Assay Kit (Thermo Fisher Scientific) and quality-controlled using Agilent RNA6000 Nano Kit on an Agilent 2100 Bioanalyzer (Agilent Technologies Japan, Ltd., Tokyo, Japan). PolyA-RNA was selected from 300 ng of total RNA, and cDNA was generated, followed by PCR amplification. cDNA Library for RNA sequencing was prepared using a NEBnext Ultra II Directional RNA Library prep with Beads (New England Biolabs Japan, Inc.). The library was quality-controlled using the Agilent 2100 Bioanalyzer and quantified using a Kapa Library Quantification Kit (NIPPON Genetics CO., Ltd.) and subjected to paired-end sequencing of 101-bp fragments using a TruSeq PE Cluster Kit v3HS (FC401-3001) on HiSeq2500 DNA sequencer (Illumina).

#### 2.4.7. Clustering and GSEA of RNAseq

First, we removed poly-A tail from 3′end and low-quality bases (quality < 30) from 5′ and 3′ end of RNA-seq reads. We also removed RNA-seq reads whose lengths are less than 30 bp. All preprocessing was performed by PRINSEQ (version 0.20.4). After preprocessing, we calculated TPM from RNA-seq data with RSEM (version 1.2.28). RSEM internally mapped RNA-seq reads to the human reference genome GRCh38 by STAR (version 2.7.1a). Then, we constructed TPM matrix where each column shows each patient, and each row shows each gene. By using TPM matrix as input, we finally performed Ward’s hierarchical clustering using Euclidean distance. Each TPM score was log2 transformed before clustering. R software (version 4.0.0) was used to perform all the statistical analyses. After the patients were clustered into two groups, the TPM matrix and the cluster labels were used to perform GSEA by GSEA software (version 4.1.0, https://www.gsea-msigdb.org/gsea/index.jsp (accessed on 1 July 2021), https://www.gsea-msigdb.org/gsea/index.jsp (accessed on 1 July 2021)).

#### 2.4.8. Genome-Wide Methylation Analysis and DKFZ Methylation Classification

For DNA methylation analysis, 500 ng of DNA extracted from frozen tumor specimen was bisulfite-modified using an EZ DNA Methylation Kit (Zymo Research, Cat.D-5002). The Infinium Methylation EPIC BeadChip Kit (Illumina, San Diego, CA, USA, hereafter EPIC array) was used to obtain genome-wide DNA methylation profiles according to the manufacturer’s instructions as previously described [28]. The raw IDAT files were uploaded to the MolecularNeuropathology website developed by the German Cancer Research Center (DKFZ)/University Hospital Heidelberg/German Consortium for Translational Cancer Research (DKTK) (the DKFZ classifier v11b4, https://www.molecularneuropathology.org/mnp? (accessed on 14 August 2021)) to obtain methylation profile-based classification and subtype scores (Table S1).

#### 2.4.9. Copy Number Alteration Analysis

Raw IDAT files from EPIC were processed using the minfi package (version 1.34.0) in R statistical environment (version 4.0.4), and quality control was performed. Mset objects generated from the raw IDAT files were used as the input data for copy number variation analysis using the conumee package (version 1.22.0). Using the genome annotations, 843,349 probes were used for further analysis. Unprocessed IDAT files of nine normal control samples were downloaded from the NCBI Gene Expression Omnibus (GEO) under the accession number GSE119776 [29]. Copy number loci proceeded by conumee package were taken as the average of each gene using R. A widely used heuristic to identify gain or loss of each gene is determined to use a symmetrical absolute cutoff of ±0.1 for conumee processed data [30].

#### 2.4.10. NanoString

The same set of genes as used by Sandmann et al. [23] for Gene Expression Subtype Classification according to Phillips et al. [31] was used for the NanoString analysis in this study (Table S2). nCounter Custom CodeSet for 31 target genes and 9 control genes was designed by NanoString. nCounter assay was performed according to the manufacturer’s instruction using 300 ng of total RNA. 

#### 2.4.11. Phillips’ Classification by NanoString

We downloaded NanoString gene expression data of GSE84010 from the GEO database as a reference. The downloaded NanoString gene expressions were labeled by three subtypes, proneural, mesenchymal, and proliferative. For each subtype, we calculated centroids of NanoString gene expression. Then, we evaluated Pearson’s correlation coefficient between the centroids of each subtype and normalized gene expressions of each patient in our BIOMARK cohort. Each patient was assigned to the subtype showing the highest correlation. Patients showing no positive correlation with any subtype were labeled as unclassified.

#### 2.4.12. Clustering of DNA Methylation Data

Beta-values of MethylationEPIC data were used for the clustering analysis of Priority 1. The EPIC probe annotations for hg38 were obtained from Zhou et al. [32]. (https://zwdzwd.github.io/InfiniumAnnotation (accessed on 7 October 2018)). Annotations excluded probes filtered out by the recommended general purpose masking, probes targeting sex chromosomes, and probes with SNPs within 5 bp from their 3′-ends. Subsequently, probes including missing values in the data of Priority 1 samples were excluded. Furthermore, the standard deviations (SDs) were calculated among the samples, and probes within the top 1% SDs were extracted. These processes left 5787 probes, which were used for the clustering analysis. The analysis was performed using R software (version 4.1.1) and gplots package (version 3.1.1). Priority 1 samples were clustered on Euclidean distances using Ward hierarchical clustering method (“ward.D2” method from hclust function.

### 2.5. Data Collection and Assessments

#### 2.5.1. Efficacy Evaluation

Efficacy was evaluated in the FAS and BBP groups, according to the Response Assessment in Neuro-Oncology Criteria for high-grade glioma [33]. A gadolinium-enhancing measurable lesion was one with a maximum perpendicular diameter of 10 mm (slice thickness of ≤5 mm). Measurements were made within 3 days after surgery and then at every 12 weeks during the maintenance phase. 

#### 2.5.2. Definitions of OS (BBP Cohort, FAS) and PFS 

OS in the BBP cohort and FAS was defined as the time (months) from the day of enrollment to death from any cause. Patients lost to follow-up were censored on the day when survival was last confirmed. PFS was defined as the time from the day of enrollment to the date of disease progression or death due to any cause. 

#### 2.5.3. Response Rate

Among patients with measurable lesions included in the efficacy analysis, the response rate was determined as the proportion of patients with a complete response (CR) or partial response (PR) after treatment.

### 2.6. Statistical Analysis

Assuming the expected 2-year survival rate of 50% and the threshold 2-year survival rate of 30%, which were derived from the AVAglio study of the Japanese patients and the entire patients in the bevacizumab arm, respectively [11], 45 patients were required in the BBP group to maintain a power ≥80% with a one-sided significance level of 5% for the 24-month registration and 24-month observation periods. Considering the ratio of patients who could start the protocol-defined secondary therapy and patient withdrawals, the total target sample size was 90 patients. Efficacy analyses were performed on the FAS and BBP groups. The Kaplan–Meier method was used to analyze survival, and the Greenwood formula was used to calculate 90% confidence intervals (CIs). Statistical methods for biomarker analysis are described in Section 2.4. The significance level was set at 5% (one-sided). Analyses of clinical data were performed using SAS version 9.4 (SAS Institute Japan Ltd., Tokyo, Japan).

## 3. Results

### 3.1. Patients

From June 2015 to December 2016, 94 patients were enrolled from 39 sites in Japan. Data cutoff was 17 January 2019, when all outcome surveys were completed, corresponding with the protocol-specified follow-up. In total, 83 patients discontinued. The major reasons were: AEs (34.0%), progression/recurrence during the second-line treatment (24.5%), and patient decline (18.1%). All 94 patients received protocol-defined primary therapy (Safety Analysis Set). Of these, 90 were diagnosed with GBM by central pathological review and were included in the FAS (Figure S2). Twenty-seven patients received protocol-defined secondary therapy (BBP), and of these, 25 without protocol deviations were included in the BBP group. Of these, 13 received either temozolomide (*n* = 12) or nimustine (*n* = 2) in combination with bevacizumab (one patient was treated with both sequentially), while the remaining 12 continued bevacizumab alone as BBP (Table S3).

The median age was 60.5 years (range, 22–75 years). Approximately half of patients (52%) had a KPS of 50–80. Most (79%) were not receiving corticosteroids at baseline. *MGMT* gene promoter methylation was observed in 33% of patients. The percentage of patients with WT *IDH1* was 93%, whereas 5% had *IDH1* mutations. Those diffuse gliomas with histological features of GBM, which were diagnosed as GBM according to the WHO Classification, 4th Ed., at the time of enrollment, have been re-classified as Astrocytoma, IDH-mutant, CNS WHO grade 4 in the latest WHO Classification, 5th Ed. (reference WHO CNS5). As such, survival analyses primarily focused on the IDH-wildtype tumors. *TERT* gene promoter mutation was observed in 66% of patients (Table 1).

### 3.2. Primary Endpoint 

#### 3.2.1. Survival: 2-Year Survival Rate

**Figure 1:**
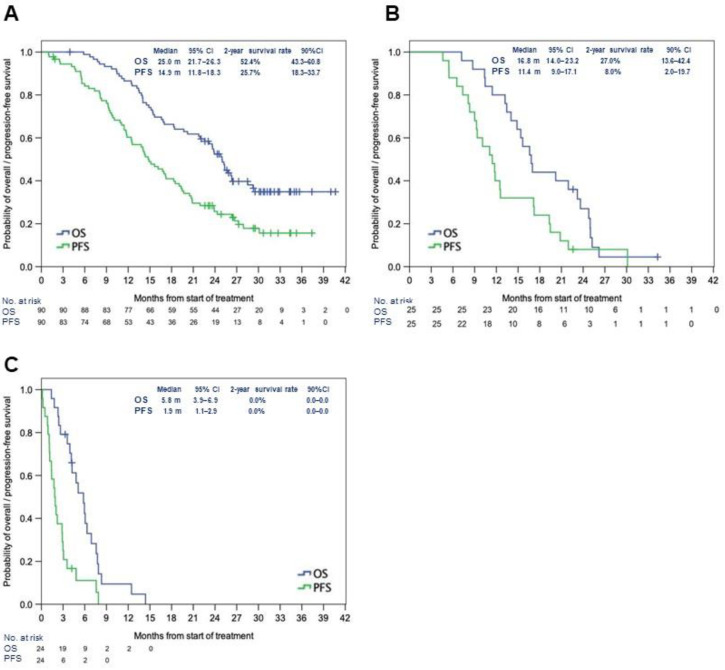
Median OS and PFS in the full analysis set (**A**), in the BBP group after initial treatment (**B**), and the BBP group after the first recurrence (**C**). Abbreviations: BBP: bevacizumab beyond progression; CI: confidence interval; mo: months; OS: overall survival; PFS: progression-free survival.

In the FAS (*n* = 90), mOS was 25.0 months (95% CI: 21.7–26.3) and mPFS was 14.9 months (95% CI: 11.8–18.3). The 2-year survival rate was 52.4% (90% CI: 43.3%–60.8%), and the 2-year PFS rate was 25.7% (90% CI: 18.3%–33.7%) (Figure 1A). In patients in the FAS solely with *IDH1*-WT GBM (*n* = 85), the mOS was 24.8 months (95% CI: 19.7–26.3) and the mPFS was 14.8 months (95% CI: 11.7–17.2) (Figure S3). In the BBP group (*n* = 25, all *IDH1*-WT), the mOS and mPFS from the initial diagnosis were 16.8 months (95% CI: 14.0–23.2) and 11.4 months (95% CI: 9.0–17.1), respectively. The 2-year survival rate was 27.0% (90% CI: 13.6%–42.4%), which did not meet the prespecified target value (50%) (primary endpoint). The 2-year PFS rate was 8.0% (90% CI: 2.0%–19.7%). In the BBP group, mOS and mPFS from the initiation of BBP were 5.8 months (95% CI: 3.9–6.9) and 1.9 months (95% CI: 1.1–2.9), respectively (Figure 1B,C). The patient background was similar between the patients in this study and those in AVAglio (Table 1) [11].

#### 3.2.2. Subgroup Analysis of the Primary Endpoint: Survival and MGMT Methylation Status

**Figure 2:**
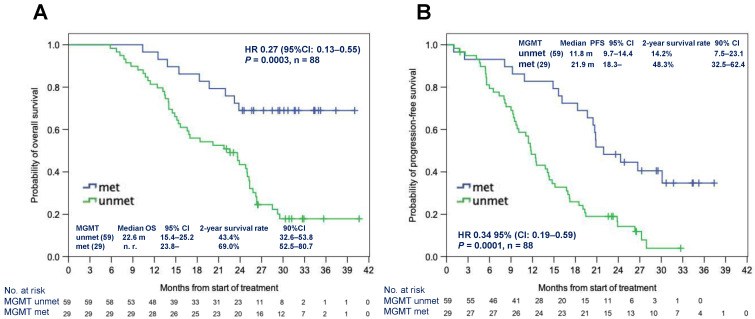
Median OS (**A**) and PFS (**B**) according to the *MGMT* methylation status in the full analysis set. Abbreviations: HR: hazard ratio; met: methylated; *MGMT*: *O*^6^-methylguanine-DNA methyltransferase; mo: months; unmet: unmethylated; OS: overall survival; PFS: progression-free survival.

Subgroup analysis using *MGMT* methylation status as a stratification factor was performed on the survival data. In the FAS, *MGMT* gene promoter was methylated in 29 patients (32%) and unmethylated in 59 patients (66%) (unknown in two patients). Patients with methylated *MGMT* had a significantly longer OS (mOS not reached vs. 22.6 months, hazard ratio [HR]: 0.27 [95% CI: 0.13–0.55], *p* = 0.0003) and PFS (mPFS 21.9 months vs. 11.8 months, HR: 0.34 [95% CI: 0.19–0.59], *p* = 0.0001) than those with unmethylated *MGMT* (Figure 2A,B). In the BBP group (*n* = 25), the *MGMT* promoter was methylated only in four (16%) patients, and it was unmethylated in 21 (84%) patients; the BBP group had a considerably higher proportion of patients with unmethylated *MGMT*. In contrast, surviving patients without progression for more than 2 years [Alive for more than 2 years with No Progression (ANP)] (*n* = 16) comprised 11 (69%) with methylated *MGMT* promoter and 5 (31%) with unmethylated *MGMT* promoter. *MGMT* methylation in the BBP group was significantly lower than in the ANP cohort (*p* = 0.001, Fisher’s exact test) (Table S4). 

### 3.3. Secondary Endpoints: Objective Response Rate, Safety

Regarding the objective response, in the FAS (*n* = 90), 39 patients who had a measurable lesion were evaluable for response; six had CR, nine had PR (i.e., overall response rate of 38.5%), twenty had stable disease (SD), and four had PD. In the BBP group (*n* = 25; 12 were evaluable), two had CR, three had PR, and seven had SD; none had PD.

Regarding safety, the protocol-defined therapies were generally well tolerated. Frequently observed AEs of special interest for bevacizumab (all grades) included hypertension (42.6%), proteinuria (29.8%), and mucocutaneous bleeding (10.6%) (Table S5). Other common AEs including myelosuppression (all grades) were lymphopenia (50%), neutropenia (27.7%), thrombocytopenia (19.1%), anemia (5.3%), appetite loss (30.9%), constipation (30.9%), nausea (18.1%), and fatigue (13.8%).

Common Grade 3 or 4 AEs were hypertension (29.8%), wound healing complications and cerebral hemorrhage (2.1%, each), and lymphopenia (41.5%). The occurrence of Grade ≥ 3 arterial thromboembolic events was 1.1% (Table S5). No new unknown toxicities were encountered.

### 3.4. Biomarker Analysis

#### 3.4.1. Methylation Classifier

When the German Cancer Research Center (DKFZ) methylation classifier was applied using methylation array data, eight patients (seven patients in the FAS and one in AVAglio) were classified as non-GBM (Priority 2, Table S1). The remaining 93 patients (78 in the FAS and 15 in AVAglio) of histologically verified *IDH1*-WT GBM were considered the biomarker cohort (Priority 1, Table S1). Among these 93 patients, nine tumors (seven in the FAS and two in AVAglio) were classified as non-neoplastic tissues, and two tumors (FAS) were unclassifiable by the DKFZ methylation classifier. These 11 tumors were diagnosed as GBM by pathology review and had mutations typically found in GBM.

#### 3.4.2. No Survival Benefit in the Proneural Subtype

To validate the findings of Sandmann et al. [23], in which *IDH1* WT proneural glioblastoma may derive an OS benefit from first-line bevacizumab treatment, we applied the gene expression classification with mesenchymal, proliferative, and proneural subtypes proposed by Phillips et al. [31] using NanoString technology [3]. All Priority 1 cases, except two cases, were successfully classified by NanoString analysis (Table S1). There were no significant differences in OS or PFS from the initial treatment among Phillips expression subtypes (Figure S4) [31]. Compared with Japanese patients with *IDH1* WT GBM enrolled in the AVAglio control arm (no bevacizumab, hereafter the “control cohort”), there were no significant differences in OS or PFS in any expression subtypes, including the proneural subtype (Figure S5).

#### 3.4.3. Novel Expression Cluster Predicted Longer Survival

**Figure 3:**
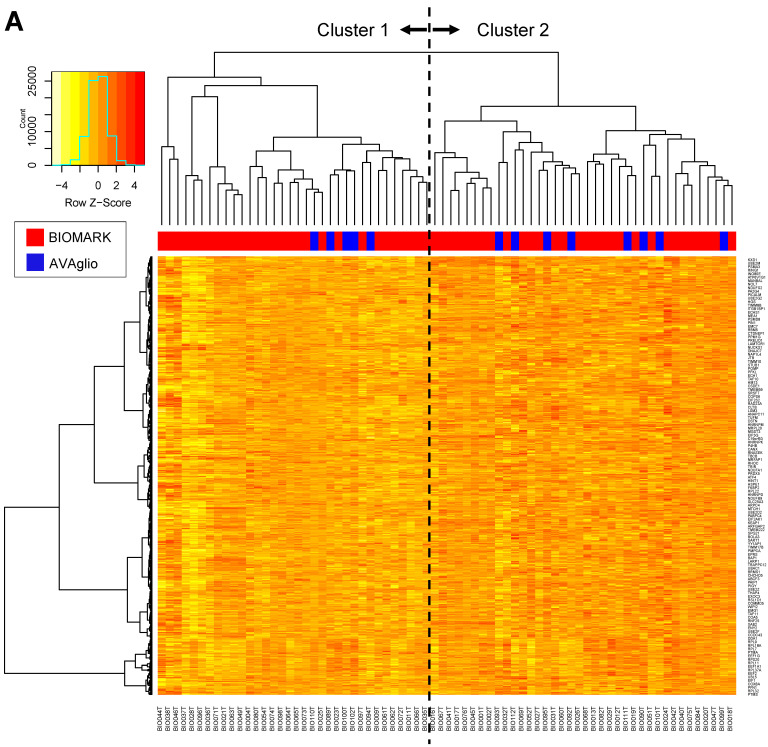

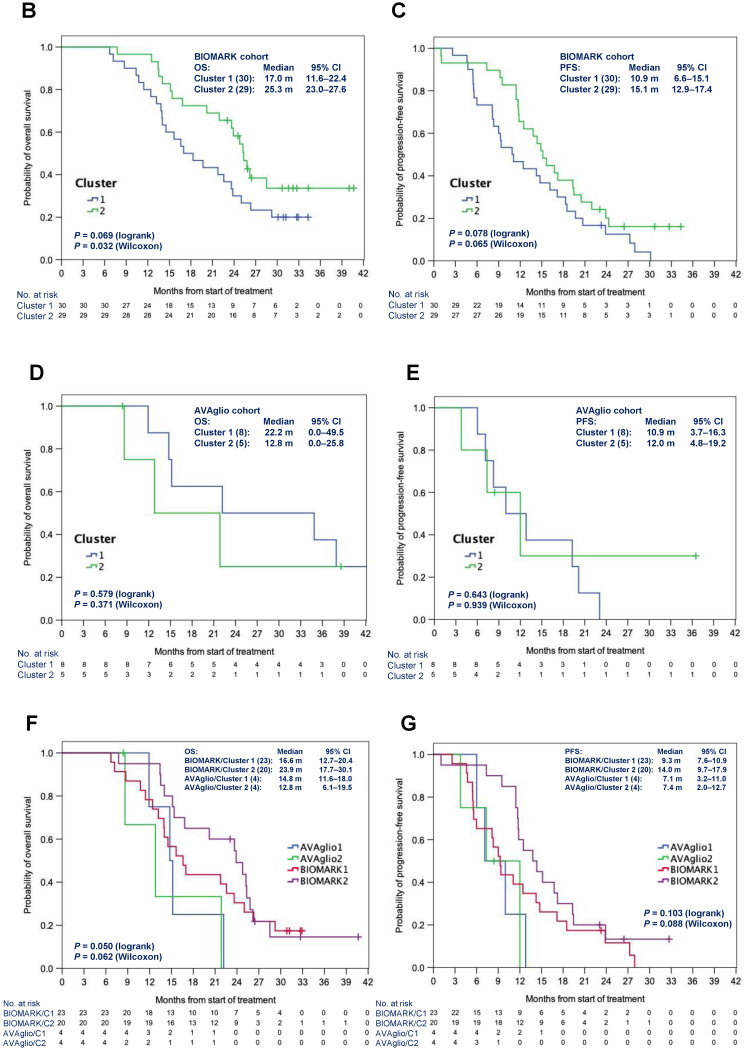
Heatmap of the 1000 genes that are most differentially expressed across the BIOMARK and AVAglio control cohorts (**A**). OS (**B**) and PFS (**C**) in the BIOMARK cohort and OS (**D**) and PFS (**E**) in the control cohort using the RNAseq Classifier. OS (**F**) and PFS (**G**) by Cluster in the BIOMARK and control cohorts with unmethylated *MGMT*.

Next, clustering analysis using the 1000 most differentially expressed genes (Top 1000 Coefficient Variance) from the RNA sequencing data of the biomarker cohort (Priority 1 including 59 BIOMARK and eight control samples) was performed. As a result, 59 patients in the BIOMARK cohort were classified into two clusters (30 in Cluster 1 and 29 in Cluster 2) (Figure 3A). Using the same condition, eight patients in the control cohort were classified in Cluster 1 and five in Cluster 2. In the BIOMARK cohort, significantly prolonged OS was observed in Cluster 2 by the Wilcoxon test (*p* = 0.032, Figure 3B). PFS tended to be longer in Cluster 2 (Wilcoxon test, *p* = 0.065) (Figure 3C). In the control cohort (*n* = 13), no difference in survival between the two clusters was observed (Figure 3D,E). When comparing survival in BIOMARK and control by cluster, OS tended to be longer (log-rank test *p* = 0.050) in Cluster 2 of BIOMARK compared with Cluster 2 of control (Figure 3F), while there were no differences in PFS (Figure 3G).

#### 3.4.4. Gene Set Enrichment Analysis Identified Distinct Expression Signatures

**Figure 4:**
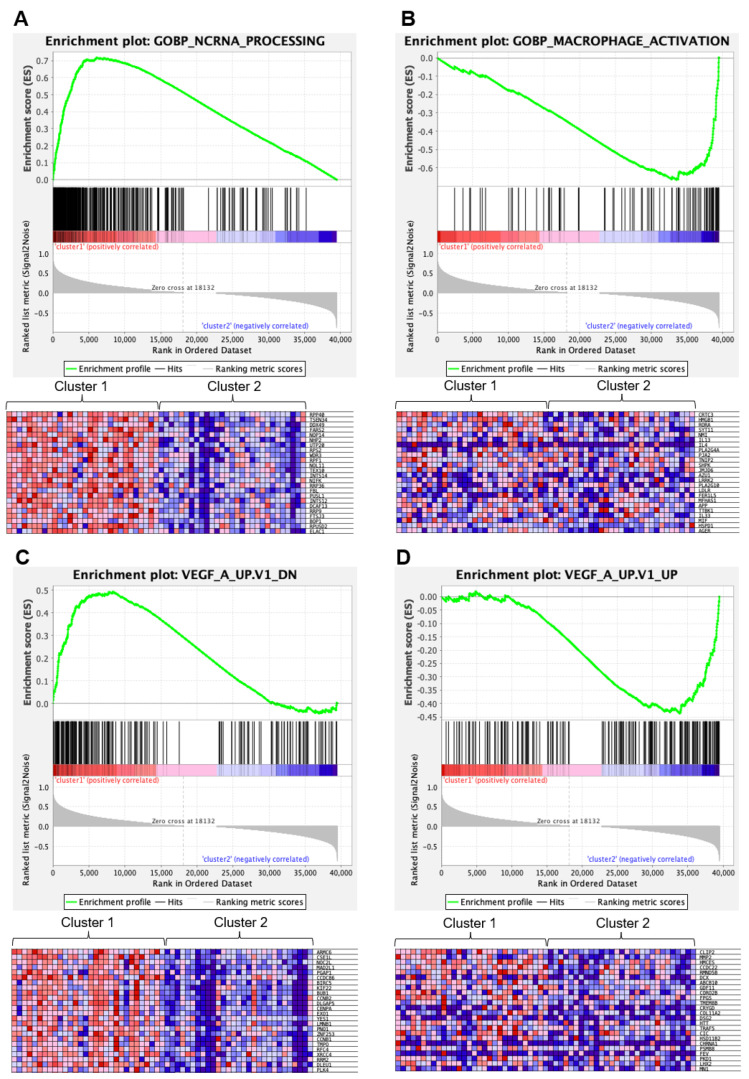
Gene Set Enrichment Analysis (Obtained through Gene Set Enrichment Analysis, https://www.gsea-msigdb.org/gsea/index.jsp (accessed on 1 July 2021)). (**A**) Top panel, enrichment plot for the Gene Set GOBP_NCRNA_PROCESSING (genes involved in any process that results in the conversion of primary non-coding RNA transcripts), enriched in Cluster 1. Bottom panel, a heat map of the clustering result using the GOBP_NCRNA_PROCESSING gene set. Only the top 24 most differentially expressed genes between Cluster 1 and 2 are shown. (**B**) Top panel, enrichment plot for the Gene Set GOBP_MACROPHAGE_ACTIVATION (genes involved in a change in morphology and behavior of a macrophage upon cytokine stimulation), enriched in Cluster 2. Bottom panel, a heatmap of the clustering result using the GOBP_MACROPHAGE_ACTIVATION gene set. Only the top 24 most differentially expressed genes between Cluster 1 and 2 are shown. (**C**) Top panel, enrichment plot for the Gene Set VEGF_A_UP.V1_DN (genes downregulated by treatment with VEGFA), enriched in Cluster 1. Bottom panel, a heatmap of the clustering result using the VEGF_A_UP.V1_DN gene set. Only the top 24 most differentially expressed genes between Cluster 1 and 2 are shown. (**D**) Top panel, enrichment plot for the Gene Set VEGF_A_UP.V1_UP (genes upregulated by treatment with VEGFA), enriched in Cluster 2. Bottom panel, a heatmap of the clustering result using the VEGF_A_UP.V1_UP gene set. Only the top 24 most differentially expressed genes between Cluster 1 and 2 are shown.

Gene Set Enrichment Analysis for the differentially expressed genes showed that Cluster 1 was enriched with genes involved in the processing and biogenesis of non-coding RNA and ribosomes, as well as telomere organization defined by Molecular Signatures Database v7.4 (http://www.gsea-msigdb.org/gsea/msigdb/index.jsp (accessed on 1 July 2021)) (Figure 4A, Figures S6 and S7, Table S6). Cluster 1 was also enriched with genes that represent signatures of the RB1 pathway (Table S7). Cluster 2 was enriched with genes involved in macrophage or microglia activation (Figure 4B, Figures S6 and S7, Table S8) and genes representing signatures of the p53 pathway or *KRAS* (Table S9). Notably, Cluster 1 was enriched with genes downregulated in endothelial cells by treatment with VEGFA, whereas Cluster 2 was enriched with genes upregulated in endothelial cells by treatment with VEGFA (Figure 4A,B; Tables S10 and S11).

#### 3.4.5. Genetic and Epigenetic Profiles

*CDKN2A* homozygous deletion was significantly more frequent in Cluster 1 (*p* = 0.037, Fisher’s exact test, Table S12). Alterations of the RB1 pathway (either *CDKN2A* homozygous deletion, *CDK4* amplification, or *RB1* mutation) and trisomy 20 were also significantly more common in Cluster 1 (*p* = 0.0251 and *p* = 0.0048). Frequencies of other molecular features examined, including *TERT* promoter mutations, *MGMT* methylation, Trisomy 7, Monosomy 10, or *EGFR* amplification, all of which are characteristic of GBM [34], were not significantly different between the two clusters (Table S12). 

Using the Cox hazard model, we performed a multivariate analysis adjusted by sex, age, and *MGMT* methylation status. Cluster 2, younger age, and methylated *MGMT* status were identified as significantly favorable independent prognostic factors for PFS. Regarding OS, methylated *MGMT* status was the only favorable independent prognostic factor (Table 2).

We also attempted to identify a novel methylation class that may predict prognosis or response to bevacizumab using the genome-wide DNA methylation array data. Clustering analysis of Priority 1 using 5624 probes (top 1% standard deviation) yielded three methylation clusters (Stratum 1–3, Figure S8). However, none of the DNA methylation strata were significantly associated with OS, PFS, or 2-year survival rates (Figure S9A,B).

## 4. Discussion

The 2-year survival rate of patients who proceeded to BBP (bevacizumab beyond progression) upon progression after initial bevacizumab-based treatment (27.0%) did not meet the prespecified criteria (50%) in this study. However, patients in the BBP group (patients who underwent BBP) were the population with early recurrence. The BBP group was enriched with patients with unmethylated *MGMT*, a well-established unfavorable prognosis factor, compared with the ANP group (patients who survived without progression) in which those with methylated *MGMT* were predominant. Patients with unmethylated *MGMT* were prone to progress earlier than those with methylated *MGMT*, explaining the low survival rate in the BBP group. Nonetheless, BBP is not recommended for use beyond the steroid-sparing effect in patients with recurrent GBM, based on the failure to demonstrate survival benefits in studies such as this [21,22]. 

Regarding safety, the frequency and severity of bevacizumab-related AEs and other events in the AVAglio study were comparable with those observed in this study (Table S5). No unexpected AEs were observed. The reason for the more frequent occurrence of Grade 3 or 4 hypertension in this study than in AVAglio is unclear, but these events did not result in other complications. 

One of the objectives was to explore biomarkers associated with the subpopulation that may respond to bevacizumab using prospectively collected fresh frozen tumor specimens to perform detailed genomic analysis. In the sub-analysis of AVAglio, patients with a proneural subtype with WT *IDH* in the bevacizumab group showed a significant improvement in OS [23]. In this study, no improvements in PFS or OS were observed in patients with the proneural subtype treated with bevacizumab. Thus, the result reported by Phillips et al. [31] was not reproduced in this study population [23]. The number of patients, especially in the control group, was considerably smaller in the current study compared with that of Sandmann et al. [23], which may explain the lack of reproducibility.

Through genome-wide gene expression profiling, we identified two novel expression classes. Significantly longer OS and a tendency for longer PFS were observed in Cluster 2 of the BIOMARK cohort. No difference was observed between the two clusters in the control cohort. This suggests that Cluster 2 may be predictive for patients who can benefit from first-line bevacizumab treatment. Cluster 1 was enriched with genes involved in ribosome biogenesis, most likely reflecting their high translational activity associated with the accelerated cell cycle. Concordantly, RB1 pathway gene alterations were significantly more common in Cluster 1, and RB1 pathway signatures were enriched in Cluster 1. Cluster 2 was enriched with genes involved in macrophage/microglial activation, presumably reflecting the increased infiltration of macrophage/microglia. Infiltrating tumor-associated macrophages and resident brain microglia (TAM) may promote the growth of GBM [35,36]. That there was no difference in the frequencies of molecular alterations characterizing GBM between the two clusters indicated that both clusters represent quintessential GBM [34]. Thus, our study identified a novel subset of bevacizumab-responsive GBM.

The most notable finding was that Cluster 1 was enriched with genes downregulated in endothelial cells by treatment with VEGFA, whereas Cluster 2 was enriched with the genes upregulated by VEGFA (https://www.gsea-msigdb.org/gsea/msigdb/ (accessed on 1 July 2021)). This suggests that Cluster 2 tumors may have been dependent on VEGFA signaling and, therefore, responsive to bevacizumab. The expression of VEGFA or VEGFR has not previously been associated with responsiveness to bevacizumab [37]. VEGFR is expressed in TAMs [38]. Considering that the expression signatures of Cluster 2 were predominantly genes associated with macrophage activation, it is likely that Cluster 2 contains high degrees of TAM infiltration. If TAMs are dependent on VEGF signaling, inhibition of VEGF signaling by bevacizumab may lead to repressing TAM-mediated promotion of GBM growth [35]. These findings should be further confirmed by histopathological investigation of the tumor specimen. For instance, the more translational activity and faster progression through the cell cycle in Cluster 1 might be reflected in more intact mitochondria per cell, more Ki-67 positive cells, or more mitotic figures in DNA/nuclear staining. Similarly, Cluster 2 might have a higher surface expression of the VEGFR and higher TAM infiltration. An extended biomarker analysis using histological specimens of the study is being planned. Although the biological basis of each cluster needs further exploration, our results may have introduced the possibility of predicting which patients could benefit from first-line bevacizumab treatment. 

This study had several limitations. It was a single-arm, uncontrolled, unrandomized study with a small number of patients and limited follow-up period, with possible bias associated with using data from another study (placebo group in AVAglio) as control data. Although the number of patients in this cohort was small, it was the only available control cohort (those GBM patients who did not receive bevacizumab) at the time. Additionally, although the study was initially aimed at evaluating the efficacy and safety of BBP, the number of patients who experienced progression after first-line radiotherapy/temozolomide/bevacizumab treatment and were enrolled in the BBP group was unexpectedly low, making the primary endpoint analysis under-powered. The number of tumors subjected to RNA sequencing was limited because of suboptimal RNA quality in some samples. The small dataset means our study should be interpreted with caution, even if collected from a prospective clinical trial. Nonetheless, we believe that the study provides a new venue to explore the true efficacy of bevacizumab in GBMs.

## 5. Conclusions

The primary endpoint of BIOMARK was not met (the 2-year survival rate in the BBP group was 27.0% vs. the target of 50%). BBP was initiated in only a small subset (27/90 patients) of the entire cohort, where *MGMT*-unmethylated tumors were predominant. We identified a novel expression cluster that may predict the prognosis of GBM patients treated with bevacizumab. Further validation of the predictive value of the novel expression classifier is warranted.

## Figures and Tables

**Table 1:** Patients’ characteristics.

Patients (%)	XXXXX	BIOMARK (*n* = 94)	AVAglio * (BEV) (*n* = 464)
Age	Median	60.5	57
	(Range)	(22–75)	(20–84)
Sex	Male	57	62
RPA class	III	17	17
	IV	7	57
	V	14	26
	Data missing	62	
KPS	50–80	52	33
	90–100	48	67
MMSE score	<27	45	24
	≥27 Data missing	49 6	76
Corticosteroids	On	79	41
	Off	21	59
GBM Histology	Confirmed	94	95
	Unconfirmed	6	5
*MGMT* status	Methylated	33	26
	Non-methylated	65	49
	Data missing	2	25
*IDH* status	IDH wildtype	93	nd
	IDH mutated	5	nd
	Data missing	2	nd
*TERT* promoter status	TERT wildtype	32	nd
	TERT mutated	66	nd
	Data missing	2	nd

(* Selected characteristics only, modified from Chinot, 2014 NEJM). Abbreviations: BEV: bevacizumab; GBM: glioblastoma; *IDH*: isocitrate dehydrogenase; KPS Karnofsky performance status; *MGMT*: *O^6^*-methylguanine-DNA methyltransferase; MMSE: mini-mental state examination; nd: not determined; RPA: recursive partitioning analysis.

**Table 2:** Multivariate analysis using Cox hazard model.

Overall Survival				
Factors	Hazard Ratio	95% CI	*p*-Value	c-Index
Sex: M/F	1.159	0.610–2.200	0.6524	0.669
Age	1.025	0.997–1.054	0.0860	0.669
*MGMT*: met/unmet	2.46	1.083–5.599	0.0316	0.669
Cluster: 1/2	0.582	0.310–1.092	0.0920	0.669
**Progression-free survival**				
Sex: M/F	1.281	0.705–2.329	0.417	0.67
Age	1.032	1.006–1.058	0.0143	0.67
*MGMT*: met/unmet	1.893	1.013–3.536	0.0455	0.67
Cluster: 1/2	0.562	0.322–0.982	0.0431	0.67

Abbreviations: CI: confidence interval; met: methylated; *MGMT*: *O^6^*-methylguanine-DNA methyltransferase; unmet: unmethylated.

## Data Availability

All raw data including FASTQ files of RNA sequences, IDAT files from the DNA methylation arrays, and BAM files from target sequencing are available from the authors upon request.

## Supplementary Materials

The following supporting information can be downloaded at: https://www.mdpi.com/article/10.3390/cancers14225522/s1, Doc S1: List of participating institutions and principal investigators.; Figure S1. BIOMARK study schema.; Figure S2: CONSORT diagram of the study.; Figure S3: Median overall survival and progression-free survival in the full analysis set solely with isocitrate dehydrogenase 1 WT glioblastoma.; Figure S4: Overall survival (OS) (A) and progression-free survival (PFS) (B) stratified by Phillips et al. subtypes: (MES) mesenchymal, (PRO) proliferative, and (PN) proneural; Figure S5: Overall survival (OS) (A, C, E) and progression-free survival (PFS) (B,D,F) of patients enrolled in BIOMARK and AVAglio (placebo group) studies stratified by Phillips et al. subtypes: mesenchymal (A,B), proliferative (C,D), and proneural (E,F).; Figure S6: Heatmap of the top 50 features for each phenotype in Cluster 1 and Cluster 2.; Figure S7: Gene Set Enrichment Analysis for genes up- or downregulated in various Gene Ontology Biological Processes.; Figure S8: Hierarchical clustering and heatmap of DNA methylation beta-values from Priority 1 samples.; Figure S9: Overall survival (OS) (A) and progression-free survival (PFS) (B) stratified by methylome stratum (1, 2, and 3).; Table S1: Biomarker analysis summary.; Table S2: Target and control genes for the NanoString analysis.; Table S3: Chemotherapeutic agent combined with BBP.; Table S4: *MGMT* promoter methylation status.; Table S5: Adverse events of special interest in patients treated with bevacizumab.; Table S6: Top 50 genes from the Gene Ontology Biological Process Gene sets enriched in Cluster 1.; Table S7: Top 50 genes from the Oncogenic Signature Gene sets enriched in Cluster 1.; Table S8: Top 50 genes from the Gene Ontology Biological Process Gene sets enriched in Cluster 2.; Table S9: Top 50 genes from the Oncogenic Signature Gene sets enriched in Cluster 2.; Table S10: Details of Gene Set Enrichment Analysis for Genes downregulated in HUVEC cells by treatment with VEGFA.; Table S11: Details of Gene Set Enrichment Analysis for Genes upregulated in HUVEC cells by treatment with VEGFA.; Table S12; Frequency of mutations and copy number alterations.
