# Supplementary material for: Bevacizumab beyond Progression for Newly Diagnosed Glioblastoma (BIOMARK): Phase II Safety, Efficacy and Biomarker Study
Source: Cancers (Basel). 2022 Nov 10;14(22):5522. doi: 10.3390/cancers14225522 (PMC9688169; doi:10.3390/cancers14225522)
Supplement: Supplementary file 1 [file cancers-14-05522-s001.zip › Nagane et al. Doc_S1_final.pdf]

Supporting Document S1

List of participating institutions and principal investigators:

We would like to thank the following institutions and investigators for their participation and contributions to this trial (alphabetical listing of the name of the institutes):

Department of Neurosurgery, Akita University Graduate School of Medicine (Toshio Sasajima)

Department of Neurosurgery, Chiba Cancer Center (Toshihiko Iuchi)

Department of Neurosurgery, Dokyo Medical University (Pyo Kim)

Department of Neurosurgery, Ehime University Graduate School of Medicine (Takanori Onishi)

Department of Neurosurgery, School of Medicine, Fujita Health University (Yuichi Hirose)

Department of Neurosurgery, Hirosaki University School of Medicine (Kenichiro Asano)

Department of Neurosurgery, Hiroshima University (Kaoru Kurisu)

Department of Neurosurgery, Hokkaido University Graduate School of Medicine (Hiroyuki Kobayashi)

Department of Neurosurgery, Iwate Medical University (Kuniaki Ogasawara)

Department of Neurosurgery, Japan Red Cross Medical Center (Yusuke Tabei)

Department of Neurosurgery, Juntendo University School of Medicine (Akihide Kondo)

Department of Neurosurgery, Graduate School of Medical and Dental Sciences, Kagoshima University (Kazunori Arita)

Department of Neurosurgery, Kanazawa University (Mitsutoshi Nakada)

Department of Neurosurgery, Kitazato University School of Medicine (Toshihiro Kumabe)

Department of Neurosurgery, Kobe University Hospital (Eiji Koumura)

Department of Neurosurgery, Kumamoto University (Hideo Nakamura)

Department of Neurosurgery, Kurume University School of Medicine (Motohiro Morioka)

Department of Neurosurgery, Kyorin University Faculty of Medicine (Motoo Nagane)

Department of Neurosurgery, Kyoto University Graduate School of Medicine (Yoshiki Arakawa)

Department of Neurosurgery, Graduate School of Medical Science, Kyoto Prefectural University of Medicine (Naoya Hashimoto)

Department of Neurosurgery, Graduate School of Medical Sciences Kyushu University (Koji Yoshimoto)

Department of Neurosurgery, Division of Clinical Neuroscience, Faculty of Medicine, University of Miyazaki (Hideo Takeshima)

Department of Neurosurgery, Graduate School of Medicine, Nagoya University (Toshihiko Wakabayashi)

Department of Neurosurgery, Nara Medical University (Ryosuke Matsuda)

Department of Neurosurgery and Neuro-Oncology, National Cancer Center Hospital (Yoshitaka Narita)

Department of Neurosurgery, Brain Research Institute, Niigata University (Yukihiko Fujii)

Department of Neurosurgery, Oita University (Minoru Fujiki)

Department of Neurological Surgery, Okayama University Graduate School of Medicine, Dentistry and Pharmaceutical Sciences (Isao Date)

Department of Neurosurgery, Graduate School of Medicine, Osaka Medical College (Yoshinaga Kajimoto)

Department of Neurosurgery, National Hospital Organization Osaka National Hospital (Toshiyuki Fujinaka)

Department of Neurosurgery, University of The Ryukyus Faculty of Medicine (Shougo Ishiuchi)

Department of Neurosurgery, Faculty of Medicine, Saga University (Tatsuya Abe)

Department of Neuro-Oncology/Neurosurgery, Saitama Medical University International Medical Center (Ryo Nishikawa)

Department of Neurosurgery, Tohoku University Graduate School of Medicine (Ryuta Saito)

Department of Neurosurgery, Graduate School of Medicine, The University of Tokyo (Akitake Mukasa)

Department of Functional Neurosurgery, Tokyo Medical Dental University (Taketoshi Maehara)

Department of Neurosurgery, University of Tsukuba Hospital (Eiichi Ishikawa)

Department of Neurological Surgery, Wakayama Medical University (Junya Fukai)

Department of Neurosurgery, Yamagata University Faculty of Medicine (Yukihiko Sonoda)
