# Supplementary material for: Bevacizumab beyond Progression for Newly Diagnosed Glioblastoma (BIOMARK): Phase II Safety, Efficacy and Biomarker Study
Source: Cancers (Basel). 2022 Nov 10;14(22):5522. doi: 10.3390/cancers14225522 (PMC9688169; doi:10.3390/cancers14225522)
Supplement: Supplementary file 1 [file cancers-14-05522-s001.zip › Nagane et al. Fig S1_final.pdf]

Figure S1

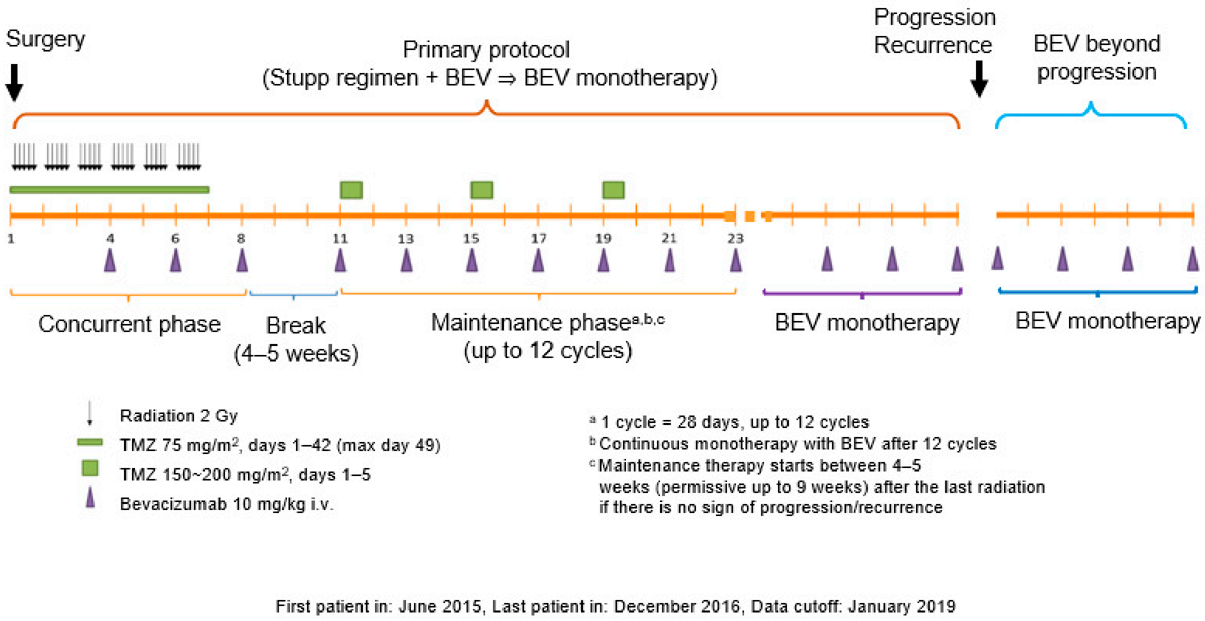

BIOMARK study schema: This study treatment consisted of two parts: the postoperative “primary therapy” and bevacizumab (BEV) beyond progression (BBP) as the “secondary therapy” given serially upon fist progression. The primary therapy included three phases: a concurrent phase (temozolomide, radiation therapy, and bevacizumab), maintenance phase (up to 12 cycles of temozolomide and bevacizumab), and monotherapy phase (bevacizumab only until progression). The secondary therapy was given as bevacizumab monotherapy or in combination with other chemotherapeutic agents.
