# Supplementary material for: Bevacizumab beyond Progression for Newly Diagnosed Glioblastoma (BIOMARK): Phase II Safety, Efficacy and Biomarker Study
Source: Cancers (Basel). 2022 Nov 10;14(22):5522. doi: 10.3390/cancers14225522 (PMC9688169; doi:10.3390/cancers14225522)
Supplement: Supplementary file 1 [file cancers-14-05522-s001.zip › Nagane et al. Fig S2_final.pdf]

Figure S2

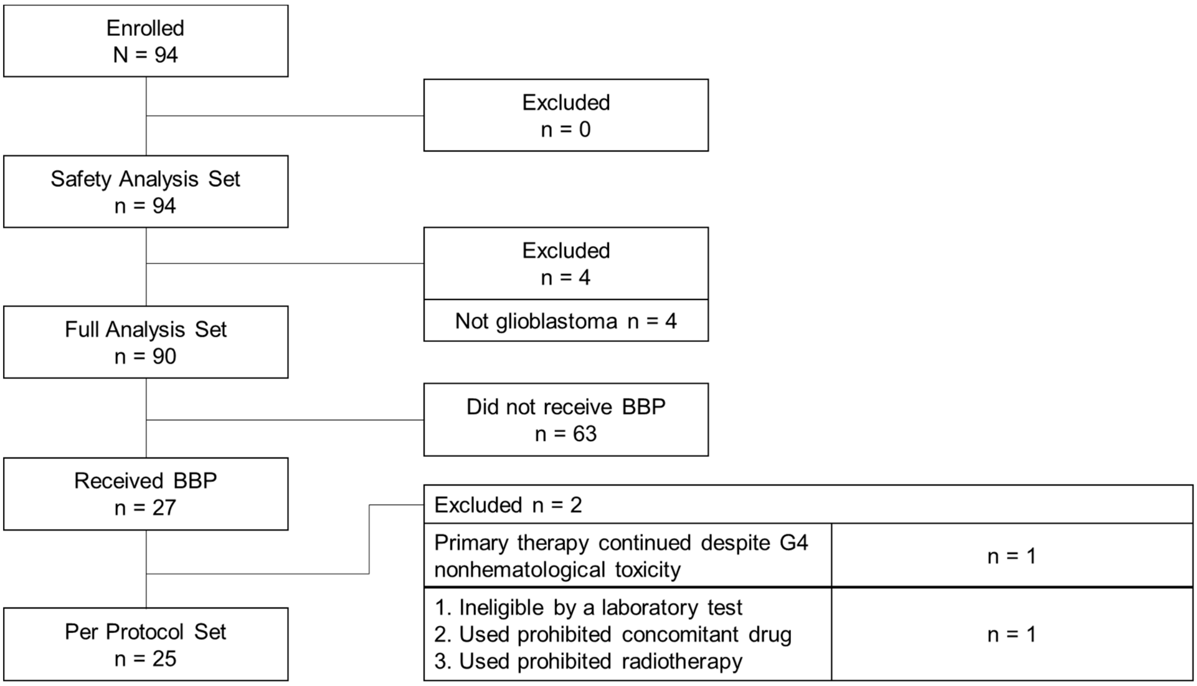

CONSORT diagram of the study: Among 94 patients who were enrolled, all 94 patients received the protocol-defined primary therapy and were included in the Safety Analysis Set. Ninety patients were diagnosed with glioblastoma by central pathological review and were included in the Full Analysis Set. Among 27 patients who received protocol-defined secondary therapy (bevacizumab beyond progression, BBP), 25 patients without protocol deviations were included in the BBP group.
