# Supplementary material for: Bevacizumab beyond Progression for Newly Diagnosed Glioblastoma (BIOMARK): Phase II Safety, Efficacy and Biomarker Study
Source: Cancers (Basel). 2022 Nov 10;14(22):5522. doi: 10.3390/cancers14225522 (PMC9688169; doi:10.3390/cancers14225522)
Supplement: Supplementary file 1 [file cancers-14-05522-s001.zip › Nagane et al. Fig S5_final.pdf]

Figure S5

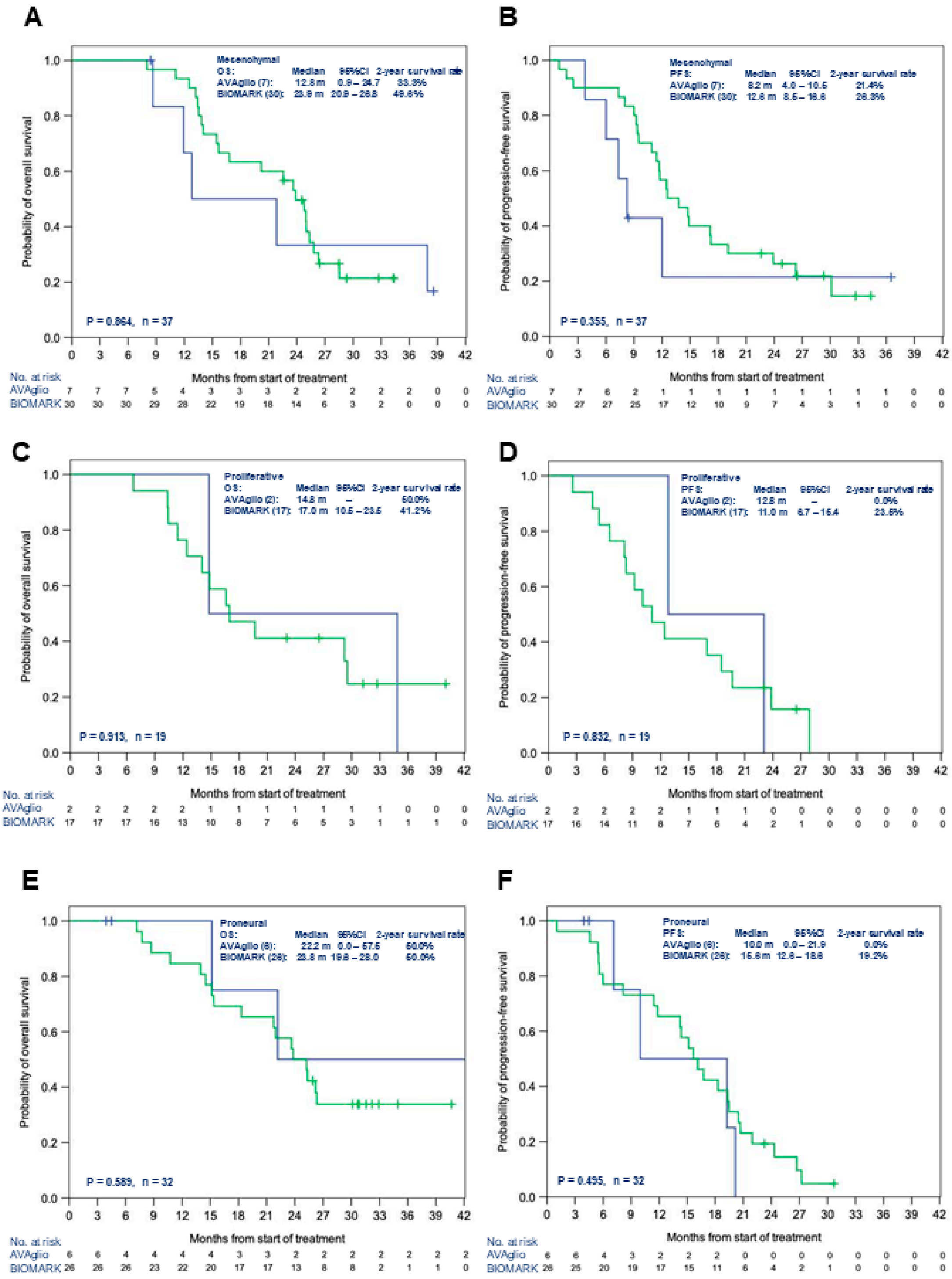

Overall survival (OS) (A, C, E) and progression-free survival (PFS) (B, D, F) of patients enrolled in BIOMARK and AVAglio (placebo group) studies stratified by Phillips et al.[1] subtypes:

mesenchymal (A, B), proliferative (C, D), and proneural (E, F). Median values with 95% confidence intervals (CIs) and 2-year survival rates are indicated for each subtype.

1. Phillips, Cancer Cell. 2006;9(3):157-73)
