# Supplementary material for: Bevacizumab beyond Progression for Newly Diagnosed Glioblastoma (BIOMARK): Phase II Safety, Efficacy and Biomarker Study
Source: Cancers (Basel). 2022 Nov 10;14(22):5522. doi: 10.3390/cancers14225522 (PMC9688169; doi:10.3390/cancers14225522)
Supplement: Supplementary file 1 [file cancers-14-05522-s001.zip › Nagane et al. Fig S6_final.pdf]

Figure S6

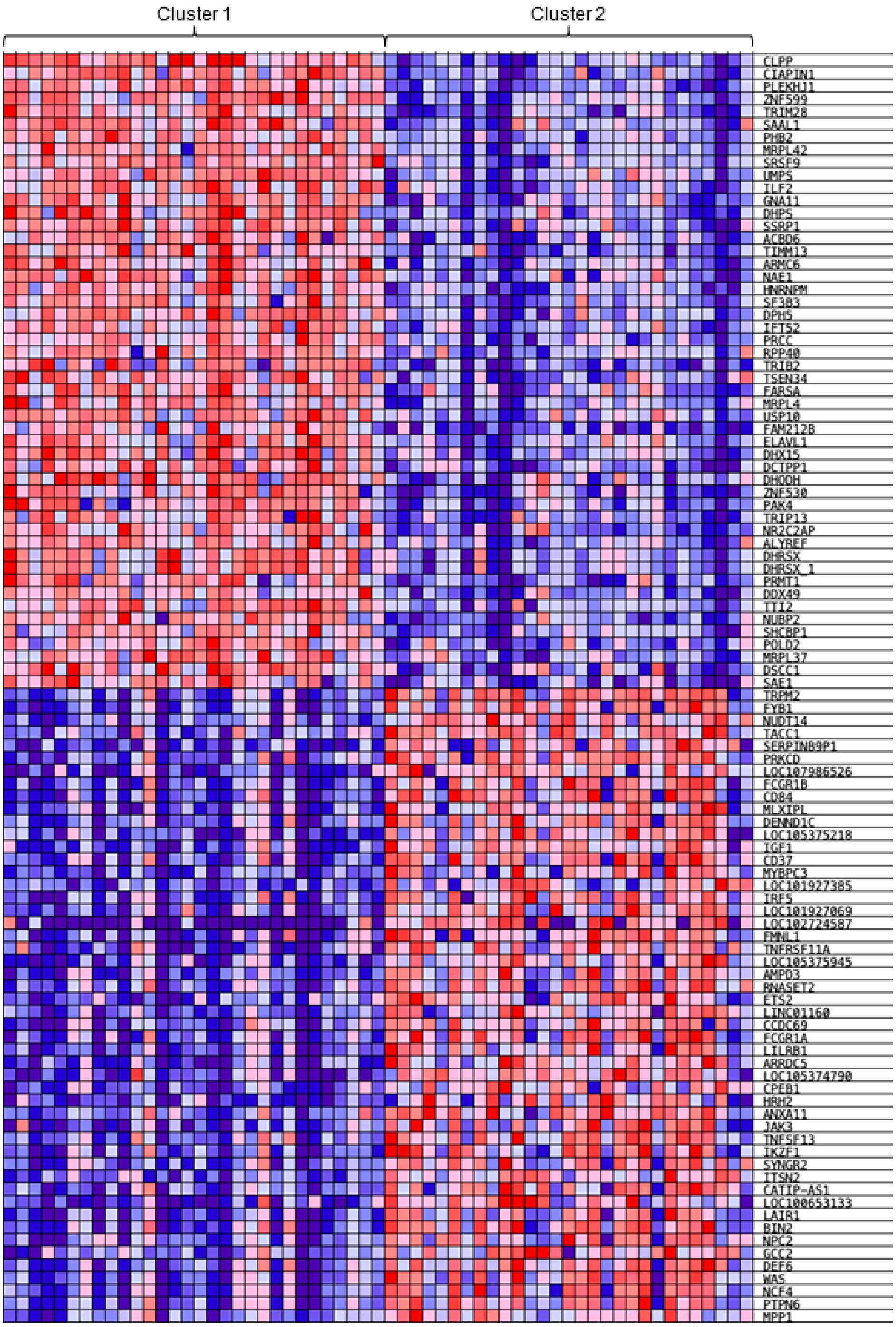

Heatmap of the top 50 features for each phenotype in Cluster 1 and Cluster 2 (Obtained through Gene Set Enrichment Analysis, https://www.gsea-msigdb.org/gsea/index.jsp).
