# Supplementary material for: Bevacizumab beyond Progression for Newly Diagnosed Glioblastoma (BIOMARK): Phase II Safety, Efficacy and Biomarker Study
Source: Cancers (Basel). 2022 Nov 10;14(22):5522. doi: 10.3390/cancers14225522 (PMC9688169; doi:10.3390/cancers14225522)
Supplement: Supplementary file 1 [file cancers-14-05522-s001.zip › Nagane et al. Fig S7_final.pdf]

Figure S7

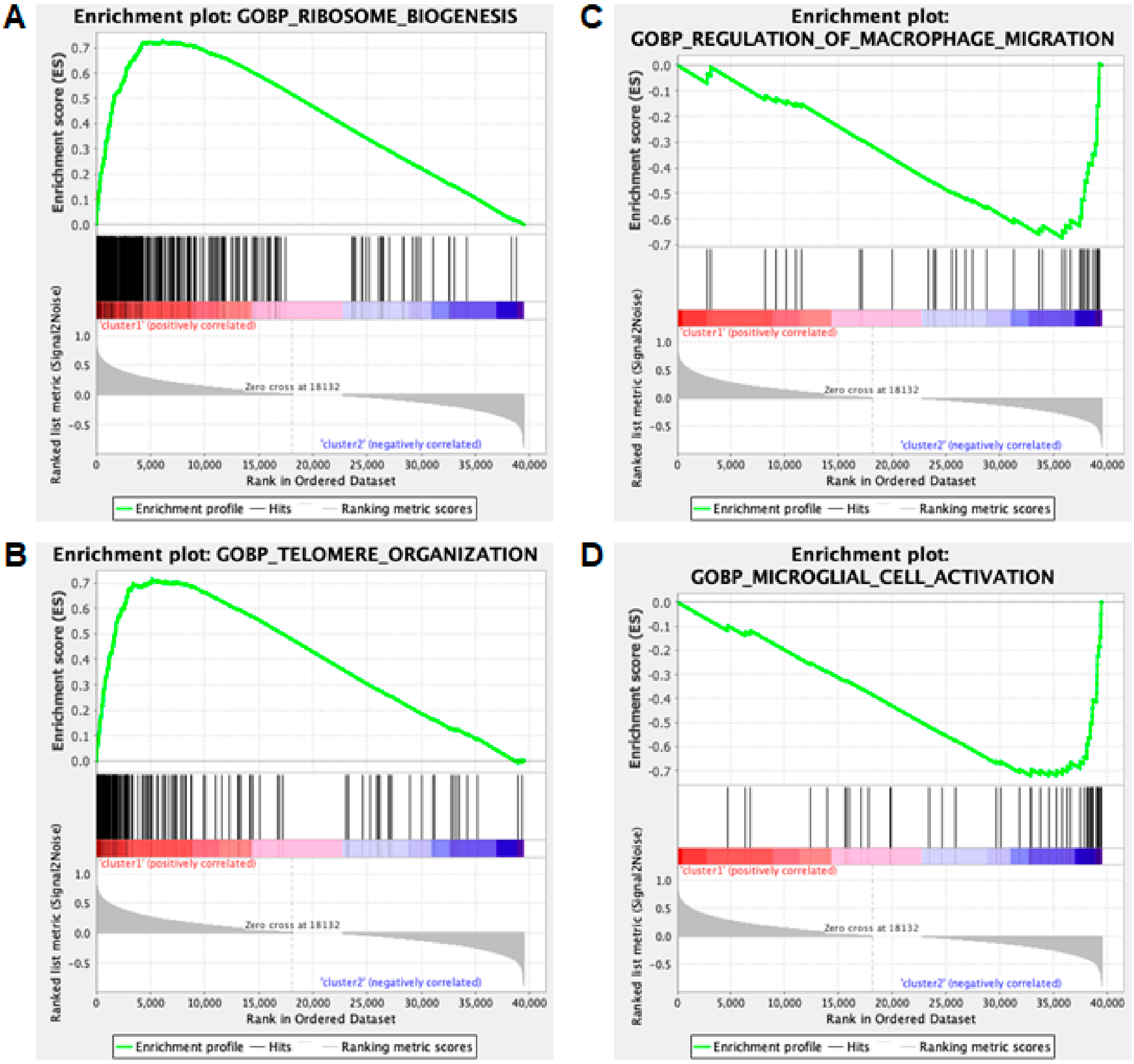

Gene Set Enrichment Analysis for genes up- or down-regulated in various Gene Ontology Biological Processes (Obtained through Gene Set Enrichment Analysis, https://www.gsea-msigdb.org/gsea/index.jsp). Enrichment plot for the Gene Set GOBP_RIBOSOME_BIOGENESIS enriched in Cluster 1 (A), the Gene Set GOBP_TELOMERE_ORGANIZATION, enriched in Cluster 1 (B), the Gene Set GOBP_REGULATION_OF_MACROPHAGE_MIGRATION, enriched in Cluster 2 (C) and the Gene Set GOBP_MICROGLIAL_CELL_ACTIVATION, enriched in Cluster 2 (D), are shown.
