# Supplementary material for: Bevacizumab beyond Progression for Newly Diagnosed Glioblastoma (BIOMARK): Phase II Safety, Efficacy and Biomarker Study
Source: Cancers (Basel). 2022 Nov 10;14(22):5522. doi: 10.3390/cancers14225522 (PMC9688169; doi:10.3390/cancers14225522)
Supplement: Supplementary file 1 [file cancers-14-05522-s001.zip › Nagane et al. Fig S8_final.pdf]

Figure S8

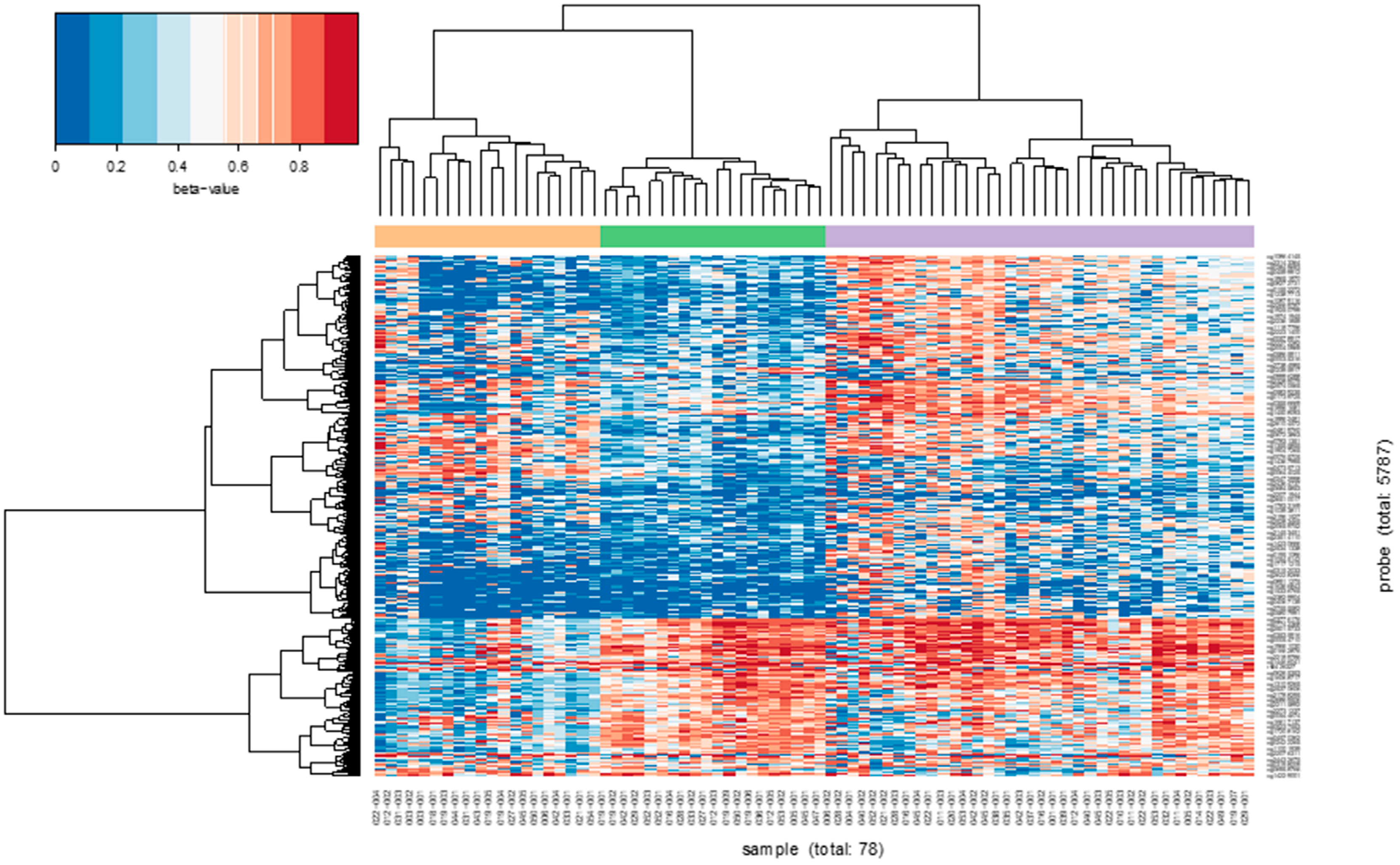

Hierarchical clustering and heatmap of DNA methylation beta-values from Priority 1 samples. After problematic probes were excluded, probes within the top highest 1% standard deviations among the samples were extracted and used for clustering. The clustering determined three methylation clusters of samples (green: stratum 1, purple: stratum 2, orange: stratum 3).
