# Supplementary material for: Bevacizumab beyond Progression for Newly Diagnosed Glioblastoma (BIOMARK): Phase II Safety, Efficacy and Biomarker Study
Source: Cancers (Basel). 2022 Nov 10;14(22):5522. doi: 10.3390/cancers14225522 (PMC9688169; doi:10.3390/cancers14225522)
Supplement: Supplementary file 1 [file cancers-14-05522-s001.zip › Nagane et al. Table_S4_final.pdf]

Table S4. *MGMT* promoter methylation status.

***MGMT* methylation**	**Methylated**	**Unmethylated**
FAS (n = 90)	29 (32%)	61 (68%)
BBP (n = 25)	4 (16%)	21 (84%)
ANP (n = 16)	11 (69%)	5 (31%)

Abbreviations: ANP, Alive >2yrs with No Progression; BBP, bevacizumab beyond progression; FAS, full analysis set; *MGMT*, O^6-methylguanine-DNA methyltransferase

P = .001, Fisher's exact test
