# Supplementary material for: Bevacizumab beyond Progression for Newly Diagnosed Glioblastoma (BIOMARK): Phase II Safety, Efficacy and Biomarker Study
Source: Cancers (Basel). 2022 Nov 10;14(22):5522. doi: 10.3390/cancers14225522 (PMC9688169; doi:10.3390/cancers14225522)
Supplement: Supplementary file 1 [file cancers-14-05522-s001.zip › Nagane et al. Table_S5_final.pdf]

Table S5. Adverse events of special interest in patients treated with bevacizumab

Adverse Events (%)	BIOMARK (N = 94)		AVAglio (N = 464)	
	All grades	Grade ≥3	All grades	Grade ≥3
Bleeding				
Cerebral hemorrhage	4.3	2.1	2.6	1.5
Mucocutaneous bleeding	10.6	0	26.7	0.4
Other	0	0	11.6	0.6
Wound-healing complications	3.2	2.1	3.7	1.5
Arterial thromboembolic events	7.4	1.1	5.0	4.1
Venous thromboembolic events	1.1	0	7.8	7.3
Hypertension	42.6	29.8	37.5	10.3
Proteinuria	29.8	1.1	14.0	3.7
GI perforation (including GI fistula/abscess)	1.1	1.1	1.7	1.1
Abscesses and fistulae	0	0	0.6	0.6
Congestive heart failure	0	0	0.4	0.4
Posterior reversible encephalopathy syndrome	0	0	–	–

Abbreviation: GI, gastrointestinal
